# Disrupted Regional Cerebral Blood Flow, Functional Activity and Connectivity in Alzheimer’s Disease: A Combined ASL Perfusion and Resting State fMRI Study

**DOI:** 10.3389/fnins.2019.00738

**Published:** 2019-07-24

**Authors:** Weimin Zheng, Bin Cui, Ying Han, Haiqing Song, Kuncheng Li, Yong He, Zhiqun Wang

**Affiliations:** ^1^Department of Radiology, Aerospace Center Hospital, Beijing, China; ^2^Department of Neurology, Xuanwu Hospital, Capital Medical University, Beijing, China; ^3^Department of Radiology, Xuanwu Hospital, Capital Medical University, Beijing, China; ^4^Beijing Key Laboratory of Magnetic Resonance Imaging and Brain Informatics, Beijing, China; ^5^State Key Laboratory of Cognitive Neuroscience and Learning, Beijing Normal University, Beijing, China; ^6^Beijing Key Laboratory of Brain Imaging and Connectomics, Beijing Normal University, Beijing, China; ^7^IDG/McGovern Institute for Brain Research, Beijing Normal University, Beijing, China

**Keywords:** regional cerebral blood flow, amplitude low frequency fluctuation, functional connectivity, arterial spin labeling, resting state fMRI, Alzheimer’s disease

## Abstract

Recent studies have demonstrated a close relationship between regional cerebral blood flow (rCBF) and resting state functional connectivity changes in normal healthy people. However, little is known about the parameter changes in the most vulnerable regions in Alzheimer’s disease (AD). Forty AD patients and 30 healthy controls participated in this study. The data of resting-state perfusion and functional magnetic resonance imaging (fMRI) was collected. By using voxel-wise arterial spin labeling (ASL) perfusion, we identified several regions of altered rCBF in AD patients. Then, by using resting state fMRI analysis, including amplitude low frequency fluctuation (ALFF) and seed-based functional connectivity, we investigated the changes of functional activity and connectivity among the identified rCBF regions. We extracted cognition-related parameters and searched for a sensitive biomarker to differentiate the AD patients from the normal controls (NC). Compared with controls, AD patients showed special disruptions in rCBF, which were mainly located in the left posterior cingulate cortex (PCC), the left and right dorsolateral prefrontal cortex (DLPFC), the left inferior parietal lobule (IPL), the right middle temporal gyrus (MTG), the left middle occipital gyrus (MOG), and the left precuneus (PCu). ALFF was performed based on the seven regions identified by the ASL method, and AD patients presented significantly decreased ALFF in the left PCC, left IPL, right MTG, left MOG, and left PCu and increased ALFF in the bilateral DLPFC. We constituted the network based on the seven regions and found that there was decreased connectivity among the identified regions in the AD patients, which predicted a disruption in the default mode network (DMN), executive control network (ECN) and visual network (VN). Furthermore, these abnormal parameters are closely associated with cognitive performances in AD patients. We combined the rCBF and ALFF value of PCC/PCu as a biomarker to differentiate the two groups and reached a sensitivity of 85.3% and a specificity of 88.5%. Our findings suggested that there was disrupted rCBF, functional activity and connectivity in specific cognition-related regions in Alzheimer’s disease, which can be used as a valuable imaging biomarker for the diagnosis of AD.

## Introduction

Alzheimer’s disease (AD) is the most common form of dementia, and it typically manifests with memory, attention, executive, visuospatial, and perceptual impairments. It is pathologically characterized by tau-related neurofibrillary tangles, amyloid-β plaques and neuronal loss (Braak and Braak, 1991). However, there is a long way to go to completely understand the neurobiology of AD, and there are no effective medications available today. In the past several years, neuroimaging studies, especially those using functional magnetic resonance imaging (fMRI), have greatly improved our comprehension of the underlying neurobiology of AD.

### ASL Perfusion Studies in AD

Arterial spin labeling (ASL) MRI can quantitatively measure regional cerebral blood flow (rCBF). By using arterial blood as an endogenous tracer, ASL shows unique advantages in ease of acquisition, radiation-free use and reliability. The perfusion pattern detected by ASL was greatly consistent with the metabolism or perfusion pattern identified by other methods, such as single photon emission computed tomography (SPECT) and positron emission tomography (PET) (Schroeter et al., 2009; Chen et al., 2011; Tosun et al., 2016; Verclytte et al., 2016). Therefore, ASL has become an increasingly promising tool as an alternative to PET and SPECT, and it shows a special potential for understanding the neural basis and early diagnosis of AD.

A majority of previous ASL studies have revealed cerebral hypoperfusion in AD, which was mainly located in the temporoparietal and posterior cingulate regions (Johnson et al., 2005; Chen et al., 2011; Le Heron et al., 2014; Liu et al., 2015; Verclytte et al., 2016; Zhang, 2016), as well as the frontal regions (Johnson et al., 2005; Chen et al., 2011; Liu et al., 2015; Verclytte et al., 2016), occipital regions (Alexopoulos et al., 2012; Zhang et al., 2013; Ding et al., 2014), basal ganglia (Grieder et al., 2013; Roquet et al., 2016), thalamus (Asllani et al., 2008; Zhang et al., 2013), and insula (Asllani et al., 2008; Grieder et al., 2013; Roquet et al., 2016). Some studies also showed hyperperfusion in several cognitive related regions, which could be explained as compensation mechanism (Alsop et al., 2008; Ding et al., 2014; Roquet et al., 2016). The detailed results were not consistent across studies due to differences in the samples, image acquisition and analytical methodology. Recently, by voxel-wise meta-analysis, an ASL study found that decreases in rCBF in AD patients were consistently revealed in the bilateral posterior cingulate cortex/precuneus (PCC/PCu), bilateral inferior parietal lobule (IPL), and left dorsolateral prefrontal cortex (DLPFC) (Ma et al., 2017).

### Resting-State fMRI Studies in AD

Recently, increasing AD studies were designed to investigate intrinsic brain activity and connectivity changes during resting state fMRI. These studies can be divided into three groups: (1) By computing regional coherence of resting state fMRI signals, some studies revealed intrinsic brain activity changes in AD patients, involving several cognitive related regions such as hippocampus, PCC/PCu and so on (Li et al., 2002; He et al., 2007; Bai et al., 2008; Wang et al., 2011). (2) By using seed based correlation analyses, many studies reported AD related disrupted functional connectivity in several specific brain regions, such as the hippocampus (Wang et al., 2006; Allen et al., 2007), PCC (Zhang et al., 2010), prefrontal cortex (Wang et al., 2007), inferior parietal lobule (Wang et al., 2015), thalamus (Wang et al., 2012), amygdala (Wang et al., 2016), basal nucleus of Meynert (Li et al., 2017), cerebellum (Zheng et al., 2017) and insula (Liu et al., 2018). (3) By using an independent component analysis (ICA) and graph-theoretical approach, several studies demonstrated the impaired large scale resting state networks in AD, involving default mode network (DMN), salience network (SN) and executive control network (ECN) (Greicius et al., 2004; Agosta et al., 2010; Dai et al., 2012). More specifically, the weaker of the DMN was closely associated with the deposition of amyloid-β plaques (Buckner et al., 2008, 2009). These intriguing studies of resting-state fMRI have advanced deep understanding of the AD pathology (Seeley et al., 2009).

Based on these previous studies, there were decreased rCBF, intrinsic brain activities and functional connectivities in the AD patients, separately. However, at present, the association between the rCBF and the intrinsic brain functional changes remains incompletely understood. Furthermore, the patterns constituted by these functional parameters in AD patients are not clear. In a recent study of healthy people, researchers combined ASL and fMRI methods to explore the association between functional connectivity strength and rCBF value during rest and memory tasks (Liang et al., 2013). The study revealed a close relationship between rCBF and brain function, suggesting the basic physiological mechanism of human brain function interaction. We speculated that the regions of decreased rCBF and brain dysfunction in AD patients might be overlapped. However, the alterations in ASL perfusion, functional activities and connectivities simultaneously in AD patients remained largely unknown.

In this study, we aimed to identify changes in rCBF, functional activities and connectivities in AD patients relative to controls. To realize this goal, we employed an integrative neuroimaging approach by combining the voxel-wise ASL and resting state fMRI methods. We first defined regions of altered rCBF in AD patients by using ASL, and then explored the intrinsic functional activity of these regions, as measured by amplitude low frequency fluctuation (ALFF). Second, we constituted networks based on the rCBF altered regions and explored the functional connectivity changes in AD patients relative to controls. Furthermore, we investigated the relationships between cognitive performances and the parameter changes. Finally, we extracted the value of rCBF and ALFF to differentiate the AD patients from the normal controls (NC) and to search for an imaging biomarker for the early diagnosis of AD.

## Materials and Methods

### Subjects

Seventy-five right-handed subjects participated in the study. During image preprocessing, five subjects (two AD patients and three NCs) were excluded because of failures in image normalization. The remaining seventy subjects, including 40 patients with AD and 30 healthy NC, participated in this study after giving written informed consent. This study was carried out in accordance with the recommendations of the Medical Research Ethics Committee of Xuanwu Hospital with written informed consent from all subjects. All subjects gave written informed consent in accordance with the Declaration of Helsinki. The protocol was approved by the Medical Research Ethics Committee of Xuanwu Hospital. The AD subjects were recruited randomly from patients who had consulted the memory clinic at Xuanwu Hospital for memory complaints. The healthy elderly controls were recruited from the local community by the recruitment advertisements. All the participants were required to complete the regular form including the age, gender, education, clinical history, family genetic history, previous examination results and so on.

All participants underwent a complete physical and neurological examination, standard laboratory tests and neuropsychological assessment. The neuropsychological examinations included the mini-mental state examination (MMSE), Montreal cognitive assessment (MoCA), auditory verbal learning test (AVLT), clock drawing test (CDT), activity of daily living scale (ADL), clinical dementia rating (CDR), Hamilton Depression Scale (HAMD), Hachinski Ischemic Score (HIS), etc. The AD patients fulfilled the new research criteria for possible or probable AD (Dubois et al., 2007, 2010).

The controls fulfilled the following criteria: (a) no visual loss or hearing loss, as well as other neurological deficiencies; (b) no stroke, depression or epilepsy, as well as other neurological or psychiatric disorders; (c) no abnormal findings in routine brain MRI (d) no complaints about cognitive and memory; (e) CDR score of 0.

The excluded criteria were as following: participants with contraindications for MRI were excluded. For example, the subjects who have a cardiac defibrillator, a pacemaker, vascular clips or a mechanical heart valve can’t take part in the examination, in addition, patients who have a history of strokes, psychiatric diseases, drug abuse, severe hypertension, systematic diseases, and intellectual disability were excluded.

### Data Acquisition

MRI examination was performed on a SIEMENS verio 3-Tesla scanner (Siemens, Erlangen, Germany). The subjects were required to hold still, keep eyes closed and think of nothing in particular. The resting state fMRI data was acquired axially using echo-planar imaging (EPI) with the following parameters: repetition time (TR) = 2000 ms, echo time (TE) = 40 ms, flip angle (FA) = 90°, field of view (FOV) = 24 cm, image matrix = 64 × 64, slice number = 33, thickness = 3 mm, gap = 1 mm, bandwidth = 2232 Hz/pixel. 3D T1-weighted magnetization-prepared rapid gradient echo (MPRAGE) sagittal images were performed with the following parameters: TR = 1900 ms, TE = 2.2 ms, inversion time (TI) = 900 ms, FA = 9°, image matrix = 256 × 256, slice number = 176, thickness = 1 mm.

Arterial spin labeling data were acquired using the following parameters: TI = 1.2 s, TI1 = 700 ms, TR = 2.0 s, TE = 14 ms, FOV = 256 × 256 mm^2^, matrix size = 64 × 64, in plane resolution = 3 × 3 mm^2^, bandwidth = 2232 Hz/px, phase partial Fourier = 6/8, EPI factor = 64. Twelve slices of 6 mm-thickness were acquired.

### rCBF Analysis

Image processing was performed using custom MATLAB (The Mathworks Inc., Natick, MA, United States) scripts and an ASL toolbox (Wang et al., 2008). The rCBF images were normalized to the Montreal neurological institute (MNI) space using the following steps: (1) Data were converted from EPI digital Imaging and Communications in Medicine (DICOM) to neuroimaging Informatics Technology Initiative (NIFTI). (2) The orientation and the origin were reset, in order to set the center to the center of each image. (3) The functional images were realigned to the first functional image of each, then M0 was registered to the mean blood oxygen level dependent (BOLD) generated during motion correction for the raw ASL images. The functional images were co-registered to the anatomical image. The mean rCBF maps were normalized into MNI space. (4) The co-registered functional images were smoothed by an isotropic Gaussian kernel with full width at half maximum (FWHM) 55 mm. (5) Batch calculation was performed for the perfusion signals.

### ALFF Analysis

Amplitude low frequency fluctuation analysis was performed using the Data Processing Assistant for Resting-State fMRI (DPARSF)^1^ (Chao-Gan and Yu-Feng, 2010). In simple terms, preprocessing included data conversion, removal of the first 10 volumes, slice timing and head motion correction. To spatially normalize the fMRI data, the realigned volumes were spatially standardized into the MNI space using the EPI template. The functional images were resampled into a voxel size of 3 × 3 × 3 mm^3^. Then, the functional images were smoothed with a Gaussian kernel of 4 mm FWHM. Finally, several nuisance covariates (twenty-four motion parameters, their first time derivations, white matter, and cerebrospinal fluid) were regressed out from the data. After preprocessing, the effects of a low frequency drift and high frequency physiological noise (respiratory and cardiac rhythms) were reduced by time bandpass filtering (0.01–0.08 Hz) of the fMRI data.

According to the rCBF results, the regions presenting with the most significant differences between the two groups were selected as cluster masks and defined as ROIs. We converted the time series of each voxel of ROIs to the frequency domain using fast Fourier transform (FFT) (parameters: taper percent = 0, FFT length = shortest), and obtained a power spectrum. We calculated the square root at each frequency of the power spectrum and obtained an average square root of 0.01–0.08 Hz per voxel, which was regarded as the ALFF, reflecting the absolute intensity of spontaneous brain activity of each voxel within ROIs at rest.

### Functional Connectivity Analysis

To explore functional connectivity changes in the AD patients, we performed a seed-based interregional correlation analysis. Based on the rCBF results, several regions showing between-group significant differences were selected as cluster masks and defined as ROIs for functional connectivity analysis. Correlation analysis between the time series of seeds was performed.

### Statistical Analyses

To assess the between-group differences of the rCBF, two-sample *t*-tests were performed, with age, gender and education level as covariates, using the Statistical Parametric Mapping software package (SPM12)^2^. The significance threshold was set to the familywise error (FWE) correction (*p* < 0.05, two tailed).

The regions that were significantly changed, in terms of the rCBF, were selected as ROIs, and independent-samples *t*-test analyses were performed to investigate the differences among the ROIs for ALFF (*P* < 0.05, SPSS20.0) and functional connectivity (*P* < 0.05, with FWE corrected) between the AD group and the NCs.

To explore the relationships among the clinical scores and the rCBF, the functional activities and the connectivities in the AD patients, a partial correlation analysis was performed with age, gender and education being used as nuisance covariates (*P* < 0.05).

Finally, we used a receiver operating characteristic (ROC) analysis with SPSS20.0 to obtain a sensitivity and specificity imaging biomarker for AD diagnosis.

## Results

### Demographic and Neuropsychological Tests

Table 1 showed demographic characteristics. We didn’t find the significant differences of gender, age and education between the AD and NC groups (both *P*s > 0.01). However, significantly lower scores of MMSE, MoCA, AVLT, CDT, and ADL were found in the AD group than that of the NC group (*P*s < 0.0001).

**TABLE 1:** Demographic and neuropsychological test.

	**AD (*n* = 40)**	**NC (*n* = 30)**	***P* Value**
Age (years)	65 ± 10	64 ± 8	0.15^*a*^
Gender (male/female)	18/22	15/15	0.51^*b*^
Education years	11.18 ± 3.19	12.58 ± 4.60	0.18^a^
CDR	0.5–2	0	–
MMSE	8–20 (14.00 ± 6.00)	26–30 (28.00 ± 2.00)	<0.001^a^
AVLT	8–24 (14.81 ± 4.12)	39–52 (44.42 ± 2.74)	<0.001^a^
MoCA	8–19 (14.94 ± 3.23)	27–30 (28.63 ± 0.67)	<0.001^a^
CDT	3–8 (6.25 ± 1.43)	8–9 (8.71 ± 0.46)	<0.001^a^
ADL	22–45 (30 ± 9.50)	20–22 (20 ± 0.30)	<0.001^a^
HAMD	0–3 (1.06 ± 1.08)	0–3 (0.61 ± 1.00)	0.07^a^
HIS	0–3 (1.16 ± 0.77)	0–3 (1.13 ± 1.07)	0.91^a^

*Data are presented as the range of minimum–maximum (mean ± SD). AD, Alzheimer’s disease; NC, normal controls; CDR, Clinical Dementia Rating; MMSE, Mini-Mental State Examination; AVLT, Auditory Verbal Learning Test; MoCA, Montreal Cognitive Assessment; CDT, Clock Drawing Task; ADL, Activity of Daily Living Scale; HAMD, Hamilton Depression Scale; HIS, Hachinski Ischemic Score; ^*a*^ The *P* value was obtained by two-sample two-tailed *t*-test; ^*b*^ The *P* value was obtained by two-tailed Pearson chi-square test.*

### Within- and Between-Group Whole Brain rCBF Changes in AD and NC

**FIGURE 1:**
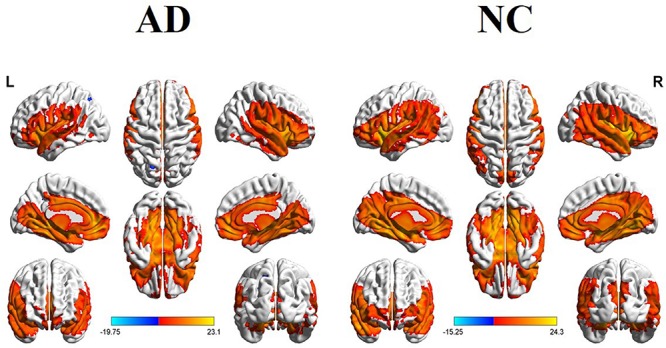
Whole brain rCBF maps in the AD and NC groups (with FEW-corrected *P* < 0.05).

Figure 1 illustrated the whole brain rCBF maps within the NC and AD groups. Visual inspection showed similar patterns between the two groups. For example, in the NC group, the increased rCBF was found in the bilateral temporal lobe, PCC/PCu, IPL, medial and lateral prefrontal cortex, middle and inferior frontal gyrus (DLPFC and IFG), occipital regions, bilateral insula, and basal ganglia regions.

**FIGURE 2:**
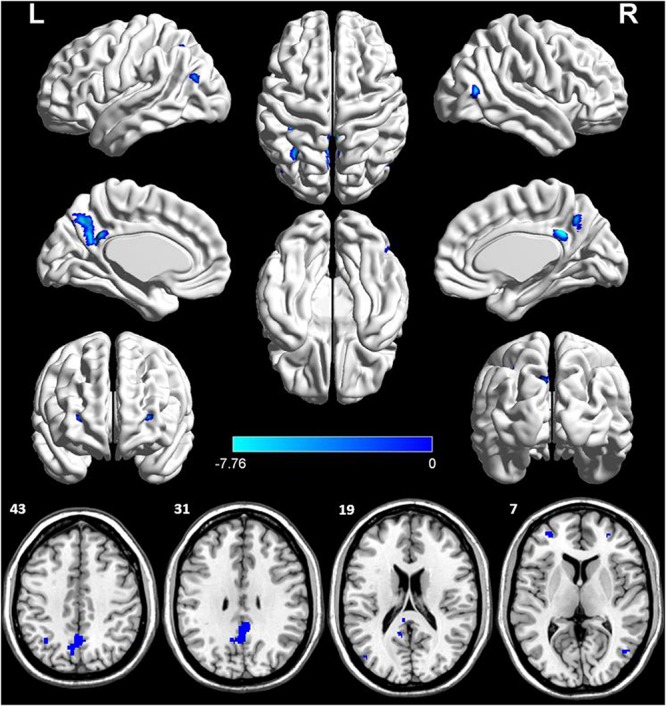
Voxel-wise percentage rCBF changes in patients with AD compared with healthy controls. Decreased rCBF in AD patients compared to healthy controls were mainly located in LPCC, LDLPFC, PDLPFC, LIPL, RMTG, LMOG, and LPCu (with FEW-corrected *P* < 0.05). L, left; R, right; PPC, posterior cingulate cortex; DLPFC, dorsolateral prefrontal cortex; IPL, inferior parietal lobule; MTG, middle temporal gyrus; MOG, middle occipital gyrus; PCu, precuneus; FEW, Familywise error.

The voxel-wise ASL analysis demonstrated that decreased rCBF in the AD patients was mainly located in the left PCC, bilateral DLPFC, left IPL, right middle temporal gyrus (MTG), left middle occipital gyrus (MOG), and left PCu. In contrast, no significant increase of rCBF was found in the AD patients relative to controls. The details were shown in Figure 2 and Table 2.

**TABLE 2:** Altered regional cerebral blood flow (rCBF) in the AD patients.

**ROIs**	**Cluster voxels**	**MNI coordinates**			**Maximum *Z***
		***x***	***y***	***z***	
L PCC	24	−6	−40	16	−6.5708
L DLPFC	23	−32	50	10	−6.1812
R DLPFC	12	28	48	6	−6.0654
L IPL	32	−34	−58	48	−6.6343
R MTG	25	46	−68	10	−6.4961
L MOG	9	−44	−72	22	−5.9128
L PCu	502	4	−46	30	−7.7593

*The significance threshold of the datas was set to Familywise error (FWE) correction (*p* < 0.05, two tailed) with age, sex and education level as covariates. L, left; R, right; PCC, posterior cingulate cortex; DLPFC, dorsolateral prefrontal cortex; IPL, inferior parietal lobule; MTG, middle temporal gyrus; MOG, middle occipital gyrus; PCu, precuneus.*

### ALFF Changes Between the AD Patients and NCs in the Resting State

**FIGURE 3:**
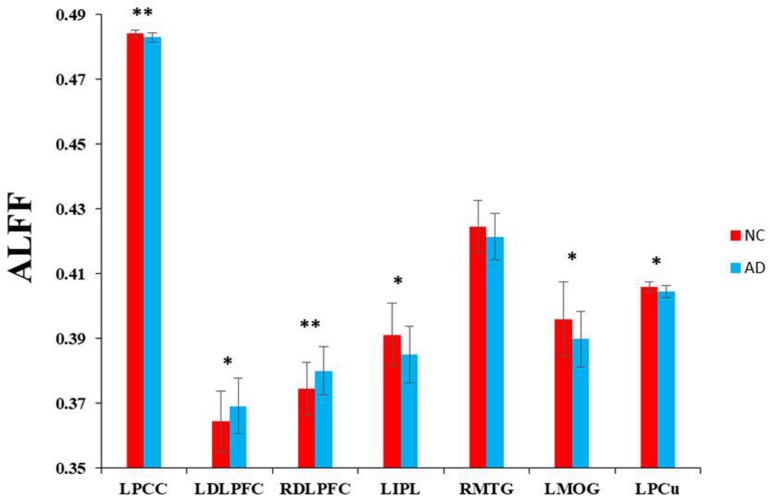
Voxel-wise ALFF changes in patients with AD compared with healthy controls in specific regions which were significantly decreased in rCBF. ^∗∗^Represents significantly changed ALFF in the AD patients compared with healthy controls (*P* < 0.01). ^*^Represents slightly changed ALFF (*P* < 0.05).

Based on the seven regions identified by the ASL method, we explored the intrinsic brain activities of each region as measured by ALFF. There were significantly decreased ALFF in the AD patients in the left PCC, left IPL, right MTG, left MOG, and left PCu. Significantly increased ALFF was found in the bilateral DLPFC (Figure 3).

### Functional Connectivity Changes Between the AD Patients and NCs in the Resting State

**FIGURE 4:**
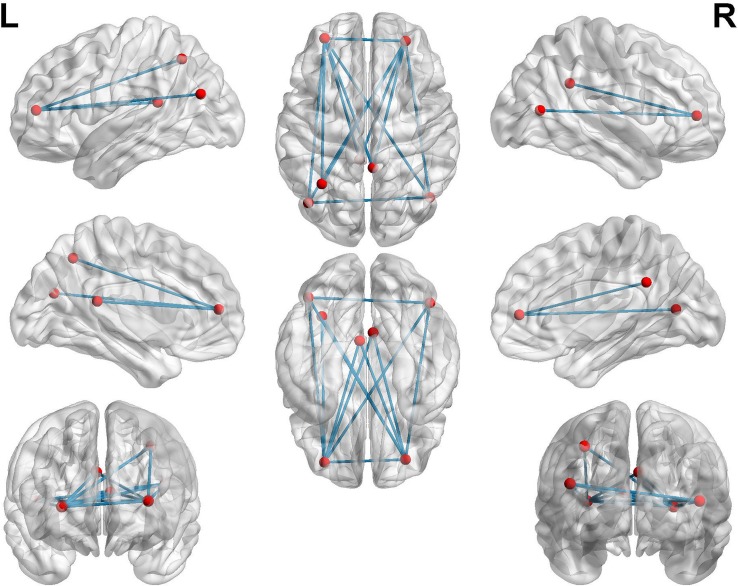
Functional connectivities alterations in the AD patients relative to controls using seed-based interregional correlation analysis.

To further investigate functional connectivity changes in the AD patients relative to controls, we selected the altered rCBF regions as seeds to perform interregional correlation analysis. Figure 4 shows decreased connectivity among the identified regions in the AD patients, predicting a disruption of the DMN, ECN, and visual network (VN).

### Relationship Between rCBF, Functional Changes and Cognitive Behaviors

**FIGURE 5:**
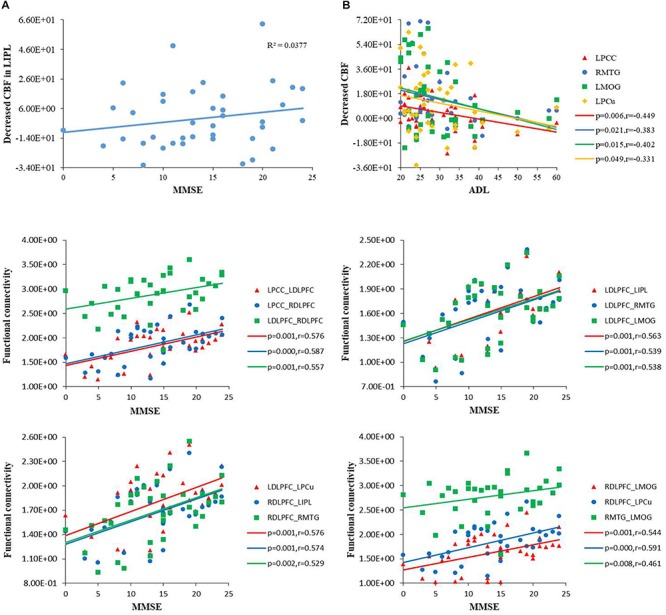
**(A)** Scatterplot of uncorrected rCBF in the IPL plotted against MMSE score, and in the PCC, MTG, MOG, PCu against ADL score. MMSE, mini-mental state examination; ADL, activity of daily living scale. **(B)** Scatterplot of functional connectivities of the DMN, ECN and VN plotted against MMSE score (*P* < 0.01). DMN, default mode network; ECN, executive control network; VN, visual network.

In the AD group, positive correlations were found between the MMSE scores and the rCBF of the IPL (*r* = 0.380, *p* = 0.022). We also found negative correlations between the ADL scores and the rCBF of several regions (i.e., PCC, MTG, MOG, and PCu). In addition, we found positive correlations between the MMSE scores and the functional connectivity of the DMN, ECN, and VN. No significant correlations were found between cognitive scores and the ALFF values. The details were shown in Figures 5A,B.

### The rCBF and ALFF Analysis of PCC/PCu as Biomarker

**FIGURE 6:**
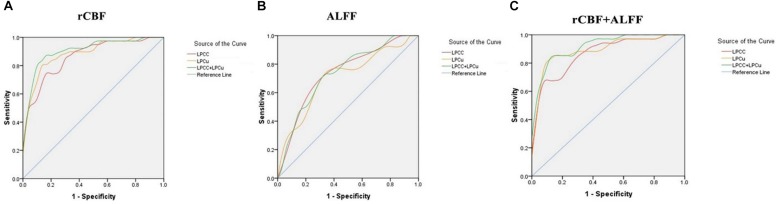
**(A)** Receiver operating characteristic (ROC) curve for uncorrected rCBF in PCC/PCu of patients with AD and healthy controls. An optimal rCBF cutoff value was determined at a sensitivity of 87.2% and specificity of 86.7%. The area under the curve (AUC) for the ROC was 0.908 (95% confidence intervals from 0.836 to 0.979). **(B)** ROC curve for ALFF in PCC/PCu of patients with AD and healthy controls. An optimal ALFF cutoff value was determined at a sensitivity of 65.7% and specificity of 73.1%. The AUC for the ROC was 0.734 (95% confidence intervals from 0.608 to 0.861). **(C)** ROC curve for uncorrected rCBF combined with ALFF in PCC/PCu of patients with AD and healthy controls. When using the two values simultaneously, it can reach a sensitivity of 85.3% and specificity of 88.5%. The AUC for the ROC was 0.921 (95% confidence intervals from 0.855 to 0.986).

Figure 6A showed the sensitivity and specificity of the rCBF of the PCC/ PCu for the AD group and controls. Using this cut-off, an rCBF value of 0.4723278, we differentiated the two groups with a sensitivity of 87.2% and specificity of 86.7%. The area under the curve (AUC) for the ROC was 0.908 (95% confidence intervals from 0.836 to 0.979). Figure 6B showed the sensitivity and specificity of the ALFF of the PCC/PCu for the AD group and controls. Using this cut-off value, 0.4044089, we differentiated the two groups by a sensitivity of 65.7% and a specificity of 73.1%. The AUC for the ROC was 0.734 (95% confidence intervals from 0.608 to 0.861). Figure 6C showed the sensitivity and specificity of the combined rCBF and ALFF of the PCC/PCu. When using the rCBF combined with ALFF of the PCC/PCu as a biomarker, it reached a sensitivity of 85.3% and specificity of 88.5%. The AUC for the ROC was 0.921 (95% confidence intervals from 0.855 to 0.986).

## Discussion

### Major Findings

To our knowledge, by combining ASL perfusion and resting state fMRI, we firstly investigated the changes of rCBF, functional activity and connectivity in the same group of AD patients. In the current study, the decreased rCBF values were found in several cognitive related regions (PCC/PCu, DLPFC, IPL, MTG, and MOG) in the AD patients, which were closely associated with the ALFF changes in the AD patients. Importantly, the AD patients presented differentially disrupted functional connectivities based on the identified rCBF regions, which were closely associated with cognitive performances. Finally, we found that the combination of rCBF and ALFF values of the PCC/PCu could be used as a sensitive biomarker to differentiate AD from controls. These findings may advance our understanding of the neural pathophysiological mechanisms of AD from perspective of neurovascular coupling.

### rCBF and ALFF Changes Between the AD Patients and NCs in the Resting State

In the current study, we found decreased rCBF in the left PCC, bilateral DLPFC, left IPL, right MTG, left MOG and left PCu in the AD patients, which was highly consistent with previous studies (Johnson et al., 2005; Chen et al., 2011; Alexopoulos et al., 2012; Zhang et al., 2013; Ding et al., 2014; Le Heron et al., 2014; Liu et al., 2015; Verclytte et al., 2016; Zhang, 2016). For example, Schroeter et al. (2009) identified a consistent hypometabolism/hypoperfusion in the posterior parietal regions, such as the PCC/PCu and IPL in the early AD patients. A reduced perfusion of the lateral prefrontal regions and temporal regions was also reported in AD studies (Johnson et al., 2005; Chen et al., 2011; Alexopoulos et al., 2012; Zhang et al., 2013; Ding et al., 2014; Liu et al., 2015; Verclytte et al., 2016). Neurovascular dysfunction, especially rCBF dysregulation and reduction, was increasingly considered to contribute to the procession of AD pathology. Specifically, a disrupted cerebral perfusion could cause impaired vascular clearance ability, which promoted deposition of Aβ and neurofibrillary tangles, leading to neurodegeneration and brain atrophy. On the other hand, the neurotoxic effects of Aβ destroyed vascular function and induced a reduced rCBF. This suggested a close relationship between vascular dysfunction and the AD Aβ pathology (Zhang et al., 2017). We can speculate that due to the vascular dysfunction in the PCC/PCu, IPL, DLPFC, MTG and MOG, the reduction in rCBF led to amyloid deposition in these regions. The early amyloid deposition in these regions further disrupted the vascular function and resulted in a reduced perfusion (Buckner et al., 2005, 2009; Grothe et al., 2016).

Additionally, we revealed a simultaneously decreased ALFF in most of the above rCBF regions, indicating a close relationship between rCBF and neuronal activity. The BOLD-fMRI signal was generated by field inhomogeneities due to the paramagnetic iron of endogenous deoxyhemoglobin (dHb) (Ogawa et al., 1990; Van de Haar et al., 2016). Therefore, MRI signal intensity changes were resulted from an altered local dHb concentration (Ogawa et al., 1990). The reduced rCBF led to an increase in the amount of dHb, and then altered the MRI signal level, which finally induced decreased neuronal activity (Logothetis, 2010). Therefore, the BOLD signal or neural activity, as measured by ALFF, reflects local changes in dHb content and depend on changes of rCBF.

### Functional Connectivity Changes Between the AD Patients and NCs in the Resting State

By exploring the functional connectivity and the network based on the altered rCBF regions, the current fMRI study revealed abnormalities in several posterior DMN regions in AD, including the PCC, the PCu, and the IPL. In the absence of tasks, DMN is the most active network (Raichle et al., 2001; Greicius et al., 2003). It is believed to be involved in the retrieval of autobiographical episodic memory and self-referential mental processes. Most neuroimaging studies showed that the structure and functional patterns of DMN in AD were destroyed, such as cortical atrophy (Dickerson and Sperling, 2009), amyloid deposition (Buckner et al., 2009), decreased regional brain activity (He et al., 2007; Wang et al., 2011) and disrupted functional connectivity (Greicius et al., 2004). Our findings provided additional evidence of decreased rCBF and disconnection of the DMN in the AD patients.

In addition to the posterior DMN regions, we also demonstrated a disconnection in the DLPFC, MTG and IPL in the AD patients, and these regions constituted the ECN. The ECN was another cognitive related intrinsic brain network involving in executive control function (Damoiseaux et al., 2006; Joo et al., 2016). By using diffusion MRI and tractography approaches, researchers demonstrated that the central IPL showed consistent fiber tracts to the dorsal frontal regions, the lateral parietal regions and the temporal regions (Caspers et al., 2011). Decreases of rCBF and disrupted connectivity of ECN regions were consistently revealed in AD patients (Agosta et al., 2012; Brier et al., 2012; Wang et al., 2015) and in individuals at high risk for AD (Liang et al., 2011). Here, we added the new evidence for the AD-related disruption of the ECN.

In addition to the above detected regions, we also observed disrupted connectivity in MOG in the AD patients. By using the fMRI method, Sala-Llonch et al. (2015) found the task related MOG activation during memory working. Another study revealed a structural disconnection in the ventral occipital-temporal cortex, suggesting failure in facial recognition (Thomas et al., 2009). Visual cognition impairment was consistently demonstrated in AD, which was attributed to the disconnection of the MOG and other regions (Cronin-Golomb, 1995; Bokde et al., 2006). Therefore, the functional disconnection between the visual cortices and other cognitive related regions in the current study may reflect a breakdown of the visual cortical network in AD patients.

To gain a deeper understanding of the correlation between rCBF and the BOLD fMRI, we noticed an increasingly recognized theory of neurovascular coupling, which suggests the association of cellular activity, rCBF, and a BOLD-fMRI signal. It was known that dynamic changes in cerebral blood flow could be affected by AD-related pathological processes. In AD, the deposition of Aβ in the cerebral vasculature, together with the impaired synapse, may lead to an attenuated BOLD response (Montagne et al., 2016), resulting in the disruption of the functional connectivity in specific cognitive- related brain regions in AD patients.

### Correlation of rCBF, Functional Changes and Cognitive Behaviors as Well as a Biomarker Analysis

In this study, we found a close relationship between cognitive impairment (MMSE and ADL) and the rCBF changes in several regions of the AD patients, which suggested that an altered rCBF in these regions can be used as an imaging marker for tracking disease progression. Furthermore, we found that MMSE scores in the AD patients were positively correlated with the functional connectivity of the DMN, ECN and VN regions, which suggested a clinical relevance of functional disconnection in the AD patients.

The AD pathological changes in the PCC/PCu have been consistently reported in previous studies (Brun and Gustafson, 1976; Braak and Braak, 1991). Disrupted activity, functional connectivity, hypometabolism and hypoperfusion in the PCC/PCu was consistently detected in all stages of AD in recent studies (Brun and Gustafson, 1976; Braak and Braak, 1991; Yoshiura et al., 2009; Sierra-Marcos, 2017). Therefore, in our study, we selected the PCC/PCu as the ROI to perform ROC analyses, aiming to identify valuable imaging biomarkers for the early diagnosis of AD. By using rCBF combined with ALFF in the PCC/PCu as a biomarker, we were able to differentiate the two groups and reached a sensitivity of 85.3%, a specificity of 88.5%, and an AUC value of 0.921 (95% confidence intervals from 0.855 to 0.986), which indicated that the combined ASL and ALFF of the PCC/ PCu were valuable imaging biomarkers for the diagnosis of early AD.

### Future Considerations

Several issues should be noticed in the future. First, our study is a cross-sectional study. Longitudinal studies are need to be performed to reveal the changes in cerebral blood flow, functional activity and connectivity with disease progression. Second, future studies of rCBF and functional connectivity changes in the prodromal and more severe stages of AD are needed, in order to clarify not only a valuable imaging biomarker for diagnosis but also a biomarker for disease severity. Third, we didn’t have gene data or other biological related recordings. In the future, we will try to design more strict experiment including the data of the gene, cerebral spinal fluid (CSF), as well as amyloid-β plaques image to perform further analysis.

## Conclusion

To our knowledge, this is the first study to investigate the changes in rCBF, functional activity and connectivity in AD patients by combining resting state BOLD fMRI and ASL techniques. In our study, we identified a disruption pattern of rCBF/ALFF/functional connectivity in the DMN, ECN and VN regions in AD patients, which showed a significant association with cognitive impairments. Furthermore, a ROC analysis demonstrated that the combination of ASL and ALFF in the PCC/PCu could be used as valuable imaging biomarker for the diagnosis of early AD.

## Data Availability

The raw data supporting the conclusions of this manuscript will be made available by the authors, without undue reservation, to any qualified researcher.

## Ethics Statement

This study was carried out in accordance with the recommendations of the Medical Research Ethics Committee of Xuanwu Hospital with written informed consent from all subjects. All subjects gave written informed consent in accordance with the Declaration of Helsinki. The protocol was approved by the Medical Research Ethics Committee of Xuanwu Hospital.

## Author Contributions

WZ wrote the manuscript. BC provided the technical support. YH and HS provided the clinical support. KL, YH, and ZW reviewed the manuscript.

## Conflict of Interest Statement

The authors declare that the research was conducted in the absence of any commercial or financial relationships that could be construed as a potential conflict of interest.

## Footnotes

1 http://rfmri.org/DPARSF

2 http://www.fil.ion.ucl.ac.uk/spm
